# Prevalence and Risk Factors Associated With Long-term Opioid Use After Injury Among Previously Opioid-Free Workers

**DOI:** 10.1001/jamanetworkopen.2019.7222

**Published:** 2019-07-17

**Authors:** Zoe Durand, Sarah Nechuta, Shanthi Krishnaswami, Eric L. Hurwitz, Melissa McPheeters

**Affiliations:** 1 Office of Informatics and Analytics, Tennessee Department of Health, Nashville; 2 University of Hawai‘i at Mānoa, Office of Public Health Studies, Honolulu; 3 Department of Medicine, Vanderbilt University Medical Center, Nashville, Tennessee; 4 Department of Health Policy, Vanderbilt University Medical Center, Nashville, Tennessee

## Abstract

**Importance:**

Using opioids for acute pain can lead to long-term use and associated morbidity and mortality. Injury has been documented as a gateway to long-term opioid use in some populations, but data are limited for injured workers.

**Objective:**

To evaluate the prevalence and risk factors of long-term opioid use after injury among workers in Tennessee who were opioid free at the time of injury.

**Design, Setting, and Participants:**

This cohort study identified injured workers aged 15 to 99 years who reported only 1 injury to the Tennessee Bureau of Workers’ Compensation from March 2013 to December 2015 and had no opioid prescription in the 60 days before injury. Participants were matched to their prescription history in Tennessee’s prescription drug monitoring program. Analysis was conducted from November 2017 to March 2018. Logistic regression models were used to calculate adjusted odds ratios (ORs) and 95% CIs for associations of demographic, injury, and opioid use variables with long-term use.

**Main Outcomes and Measures:**

The primary outcome was long-term opioid use, defined as having an opioid supplied for 45 or more days in the 90 days after injury.

**Results:**

Among 58 278 injured workers who received opioids after injury (18 977 [32.5%] aged 15-34 years, 27 514 [47.2%] aged 35-54 years, and 11 787 [20.2%] aged 55-99 years; 32 607 [56.0%] men), 46 399 (79.6%) were opioid free at the time of injury. Among opioid-free injured workers, 1843 (4.0%) began long-term opioid use. After controlling for covariates, long-term use was associated with receiving 20 or more days’ supply in the initial opioid prescription compared with receiving less than 5 days’ supply (OR, 28.94; 95% CI, 23.44-35.72) and visiting 3 or more prescribers in the 90 days after injury compared with visiting 1 prescriber (OR, 14.91; 95% CI, 12.15-18.29). However, even just 5 days’ to 9 days’ supply was associated with an increase in the odds of long-term use compared with less than 5 days’ supply (OR, 1.83; 95% CI, 1.56-2.14).

**Conclusions and Relevance:**

In this study of injured workers, injury was associated with long-term opioid use. The number of days’ supply of the initial opioid prescription was the strongest risk factor of developing long-term use, highlighting the importance of careful prescribing for initial opioid prescriptions.

## Key Points

**Question:**

What is the prevalence of long-term opioid use after injury among previously opioid-free workers in Tennessee, and what risk factors are associated with developing long-term opioid use after injury?

**Findings:**

In this cohort study of 46 399 injured workers who were opioid free at the time of injury, 4.0% had long-term opioid use after injury. Long-term use was associated with receiving 20 or more days’ supply in the initial opioid prescription and visiting 3 or more prescribers.

**Meaning:**

Prescribing characteristics were the strongest risk factors for long-term opioid use after injury.

## Teaser

This cohort study evaluates the prevalence and risk factors of long-term opioid use after injury among workers in Tennessee who were opioid free at the time of injury.

## Introduction

Long-term opioid use is about more than the number of days someone takes an opioid; it is also about the threshold at which the costs of opioid use begin to outweigh the advantages. Research on long-term opioid use for chronic pain was summarized in a systematic review of clinical trials and observational studies,^1^ which reported no evidence of effectiveness for opioid use of greater than 3 months but increased risk of harms, including overdose, substance use disorder, fractures, and myocardial infarction. An epidemiological study^2^ showed that long-term opioid use is associated with dependence, addiction, poor self-rated health, inactivity, unemployment, higher health care utilization, and poor self-rated quality of life. In injured workers specifically, long-term opioid therapy is associated with higher health care costs^3^ and lower productivity.^4^

A 2018 study of workers’ compensation (WC) claimants in Maryland^5^ examined factors associated with opioid use more than 90 days after injury, focusing on patient characteristics. Associations with opioid use after 90 days were found for older age (≥60 years vs 15-29 years: odds ratio [OR], 1.92; 95% CI, 1.56-2.36), higher annual income (≥$60 000 vs <$20 000: OR, 1.31; 95% CI, 1.07-1.61), crush injuries vs soft-tissue or contusion injuries (OR, 1.55; 95% CI, 1.28-1.89), strains and sprains vs soft-tissue or contusion injuries (OR, 1.54; 95% CI, 1.36-1.75), and chronic joint pain (OR, 1.98, 95% CI 1.79-2.20).^5^

A 2017 study of a commercially insured population^6^ pointed to the number of days’ supply of the initial prescription as the single largest factor in continued opioid therapy for more than 1 year and more than 2 years after age, sex, mental comorbidities, and dose were controlled for. A retrospective cohort study conducted in Oregon using prescription drug monitoring program data^7^ similarly reported strong associations of dose (400-799 morphine milligram equivalents [MME] vs <120 MME in a month: OR, 2.96; 95% CI, 2.81-3.11) and number of prescriptions filled (2 fills/mo vs 1 fill/mo: OR, 2.25; 95% CI, 2.17-2.33) with risk of long-term use of opioids (defined as ≥6 fills/y after initiation) among previously opioid-free patients. Another study in Utah^8^ found increased odds of long-term opioid use (defined as ≥120 prescription days or ≥90 prescription days with ≥10 fills in a year) in patients who received benzodiazepines in the first 14 days of care (OR, 1.87; 95% CI, 1.01-3.48). Among injured workers with back injuries, higher dose in the first 3 months after injury has been shown to be associated with long-term opioid use even after baseline pain and injury severity are controlled for (900-1799 MME vs ≤899 MME in first 3 months after injury: OR, 4.01; 95% CI, 2.23-7.20).^9^

Following the US Centers for Disease Control and Prevention guidelines, providing the lowest dose of a short-acting opioid analgesic for the fewest days possible (preferably 3 and no more than 7 days) when opioids are initiated may mitigate the risk of developing long-term use^10^; however, data are limited on the association of prescription-based factors, such as days’ supply and dose, with long-term use among injured workers. This cohort study was conducted to develop a predictive model for the development of long-term opioid use in previously opioid-free injured workers. Demographic and prescribing characteristics hypothesized to be factors associated with long-term use were examined in previously opioid-free injured workers using Tennessee’s Controlled Substances Monitoring Database (CSMD).

## Methods

This study was approved by the institutional review boards at the Tennessee Department of Health and University of Hawaii and follows the Strengthening the Reporting of Observational Studies in Epidemiology (STROBE) reporting guideline for cohort studies.^11^ This study had no contact with participants and was exempt from collecting informed consent.

### Study Design and Population

A detailed description of cohort creation was previously published.^12^ Briefly, data on workplace injuries from Tennessee WC were linked to prescription records in the CSMD. Injured worker data were obtained from WC records (First Report of Injury, shared by the Tennessee Department of Labor), which is required regardless of the employee’s intention to pursue medical care or a WC claim.^13^ The CSMD contains data on prescriptions for controlled substances that are filled, and Tennessee dispensers are generally required to report filled prescriptions within 1 business day, with the exception of veterinarians, who were excluded from this study.^14^

Data cleaning (eg, correcting errors and standardizing fields to enable linkage and analysis) and linkage between data sources on name and date of birth has been previously described.^12^ To allow for prescriptions to be measured from 60 days prior to injury through 180 days after injury, prescription records were accessed from January 1, 2013, to June 30, 2016, and WC records were accessed from March 2, 2013, to December 31, 2015. Analysis was conducted from November 2017 to March 2018.

The main study population was opioid free at the time of injury, defined as having no record of receiving an opioid prescription for 60 days before the injury.^15^ Opioid dispensing in Tennessee is limited to 30 days’ supply,^16^ and a 60-day restriction allows for only 2 consecutive periods of 30-day prescriptions. No injured workers receiving opioids for medication-assisted treatment were included in the study. Eligibility required complete name and date of birth data in the WC record to enable matching to the CSMD, having sex data, having a physical injury, and being aged 15 to 99 years. To avoid the confounding effect of multiple injuries, eligibility was restricted to injured workers who reported only 1 injury during the study period. Opioid prescriptions were included if they were opioid class and schedule 2 to 4 (schedule 5 was excluded owing to very low dose, eg, cough syrups) and excluded if they were known to be dispensed by a veterinarian or Veterans Affairs pharmacy. Veterans Affairs pharmacies had incomplete reporting, composed a low amount (1%) of all opioid prescriptions, and were excluded owing to concerns regarding missing data. Prescription criteria resulted in only prescriptions being excluded, not patients. Opioid prescriptions were measured from 60 days before each person’s date of injury (earliest January 1, 2013) to 180 days after each person’s injury (latest June 30, 2016).

### Demographic Characteristics and Clinical Information

Age at the time of injury, sex, marital status, type of injury, part of body injured, and residence type were selected from WC records on the basis of availability, completeness, and previous literature.^7,17,18^ Type of injury was categorized into strains, sprains, and tears; fractures; and other on the basis of frequency and hypothesized association with long-term opioid use. Part of body injured was categorized into lower back, finger(s), knee, and other on the basis of frequency and association with long-term use. Additional clinical data were not available. Residence type was identified from zip codes and classified as urban (residing in a county with one of Tennessee’s 6 largest cities) or rural (residing in one of the other 89 counties).

Postinjury opioids were classified by type, formulation (short-acting or long-acting), payment (cash or other), and overlapping days with a benzodiazepine prescription. Maximum daily dose in MME and maximum days’ supply were identified. Each prescription measure, including dose and days’ supply, was completed for 7 days, 30 days, and 90 days after injury. Additional prescription characteristics included dose and days’ supply of the first prescription, receiving benzodiazepine in the 60 days before the injury, and the number of prescribers and pharmacies visited during the 90 days after the injury. For workers who received multiple opioid prescriptions on the same day (1695 [2.9%]), MME and days’ supply were summed for all eligible opioid prescriptions received on that day.

### Primary Outcome

Long-term opioid analgesic use, the primary study outcome, was measured with prescription days, ie, the days’ supply of an opioid prescription added to the date on which the opioid was received. For overlapping prescriptions, prescription days were only counted once. Corresponding to the Centers for Disease Control and Prevention definition of chronic pain as lasting longer than 3 months,^19^ long-term use was defined as receiving an opioid on most days for a 90-day period, measured as 45 or more prescription days in 90 days after injury. Nonoverlapping prescription days were summed for all prescriptions that were received and did not need to be continuous.

### Statistical Analysis

A split data set approach^20^ was used for model building. The sample of opioid-free injured workers whose injuries occurred in 2013 and 2014 (n = 30 608) was used for the derivation model, and those with injuries in 2015 (n = 15 791) were used for validating the derivation model. Numbers and percentages were used to describe the distribution of demographic and clinical characteristics in the population by opioid-free status.

To visualize the arc of opioid use over time, the number of injured workers who received opioids on most days was calculated by 30-day periods from 0 to 180 days after injury and graphed. To identify associations with long-term opioid use, unconditional logistic regression was used to compute unadjusted and adjusted ORs with 95% CIs for the binary outcome.

To build the derivation model, demographic and clinical variables that showed an association with long-term use in unadjusted models were selected for inclusion in a multivariable model. Where different time frames had been considered for opioid-use measures (eg, long-acting opioid received within 7 days vs 30 days vs 90 days of injury), the time frame with the highest associated point estimate was included in the starting multivariable model. The multivariable model was then refined with feedback from receiver operating characteristic curve C statistics,^21^ systematically changing variables and time frames to maximize model discrimination. Model fit was assessed based on deviance statistics, Pearson statistics, and Hosmer-Lemeshow statistics. After the best-fitting derivation model had been achieved, the model parameters were applied to the validation group to form the validation model, and the validation model fit and associations were compared with the derivation model. SAS version 9.4 (SAS Institute) was used for all analyses. *P* < .05 was considered statistically significant, and all tests were 2-tailed.

## Results

### Demographic Characteristics and Opioid-Free Status

**Figure 1.**
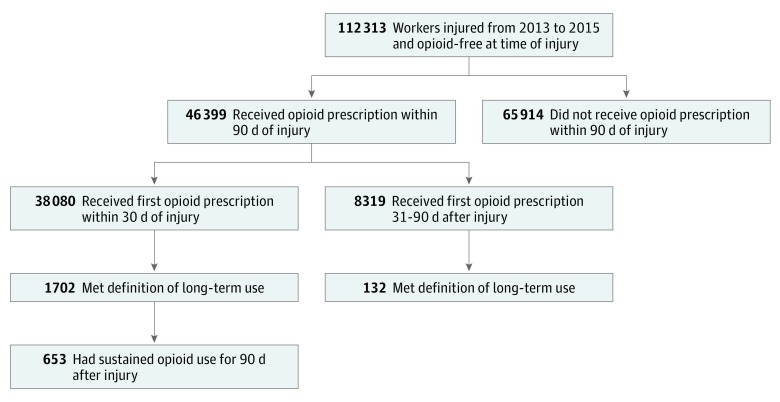
Flowchart of Opioid Receipt and Use After Injury Among Previously Opioid-Free Workers Long-term use was defined as having an opioid supplied for 45 or more days in the 90 days after injury. Sustained use was defined as having received opioids on most days in all 30-day periods after injury (ie, 0-30 days, 31-60 days, and 61-90 days).

Of 205 565 injured workers who reported 1 injury to Tennessee WC, 128 885 met eligibility criteria, and 58 278 received opioids in the 90 days after injury (18 977 [32.5%] aged 15-34 years, 27 514 [47.2%] aged 35-54 years, and 11 787 [20.2%] aged 55-99 years; 32 607 [56.0%] men). Among injured workers who received opioids, 46 399 (79.6%) were opioid free at the time of injury (Figure 1 and Table 1). Most (38 080 [82.1%]) received their first opioid prescription within a month of injury, but 8319 (17.9%) received their first opioid prescription later. Overall, 1834 (4.0%) of those who received opioids began long-term use. Sustained opioid use (ie, received an opioid on most days for every 30-day period up to 90 days after injury) was found in 653 patients (1.7%). The highest dose received in the first prescription by an opioid-free patient was 770 MME.

**Table 1.** Sample Selection Method and Description of Sample Size

Study Selection Criteria	No.	
	Derivation Model (March 2, 2013-December 31, 2014)	Validation Model (January 1, 2015-December 31, 2015)
Reported only 1 physical injury to Tennessee Workers’ Compensation, 2013-2015	133 309	72 256
Able to be linked to prescription history (not missing name, sex, or date of birth)	132 299	71 836
Aged 15-99 y	131 882	71 672
Resided in Tennessee	83 853	45 032
Received an opioid within 90 d of injury	38 646	19 632
Opioid free at time of injury and included in analysis	30 608	15 791

Injured workers who started using opioids late (received first opioid prescription 31-90 days after injury) had a similar age distribution as those who started using opioids early (received first opioid within 30 days of injury) but were significantly more likely to be female (15 460 [48.9%] vs 4066 [40.6%]) and have back injuries (4760 [12.5%] vs 773 [9.3%]) and were less likely to have fractures (914 [2.0%] vs 915 [11.0%]). Compared with injured workers with a recent history of opioid use, opioid-free injured workers in the derivation model were significantly more likely to be younger than 35 years (10 663 [34.8%] vs 1925 [24.0%]), male (17 687 [57.8%] vs 3921 [48.8%]), have a fracture (2755 [9.0%] vs 439 [5.5%]), have a finger injury (3118 [10.2%] vs 603 [7.5%]), and be from urban areas (11 681 [38.2%] vs 2548 [31.7%]) (Table 2). The population used for the validation model showed similar distributions.

**Table 2.** Demographic and Clinical Characteristics of 58 278 Injured Workers Who Reported 1 Injury to Tennessee Workers’ Compensation From March 2, 2013, to December 31, 2015, and Received Opioids Within 90 Days of Injury, by Opioid-Free Status at Time of Injury

Characteristic	Opioid Free, No. (%)			
	Derivation Model		Validation Model	
	No (n = 8038)	Yes (n = 30 608)	No (n = 3841)	Yes (n = 15 791)
Age, y				
15-34	1925 (24.0)	10 663 (34.8)	839 (21.8)	5550 (35.2)
35-54	4278 (53.2)	14 121 (46.1)	2041 (53.1)	7074 (44.8)
55-99	1835 (22.8)	5824 (19.0)	961 (25.0)	3167 (20.1)
Sex				
Male	3921 (48.8)	17 687 (57.8)	1826 (47.5)	9173 (58.1)
Female	4117 (51.2)	12 921 (42.2)	2015 (52.5)	6618 (41.9)
Marital status				
Single	188 (2.3)	694 (2.3)	88 (2.3)	303 (1.9)
Married	2403 (29.9)	8749 (28.6)	1079 (28.1)	4240 (26.9)
Widowed, separated, or divorced	1796 (22.3)	6561 (21.4)	1012 (26.4)	3841 (24.3)
Missing/unknown	3651 (45.4)	14 604 (47.7)	1662 (43.3)	7407 (46.9)
Type of injury				
Strain, sprain, or tear	3193 (39.7)	11 994 (39.2)	1480 (38.5)	5839 (37.0)
Fracture	439 (5.5)	2755 (9.0)	214 (5.6)	1596 (10.1)
Other	4406 (54.8)	15 859 (51.8)	2147 (55.9)	8356 (52.9)
Part of body injured				
Lower back	965 (12.0)	3723 (12.2)	458 (11.9)	1791 (11.3)
Finger(s)	603 (7.5)	3118 (10.2)	279 (7.3)	1524 (9.7)
Other	6470 (80.5)	23 767 (77.7)	3104 (80.8)	12 476 (79.0)
Residence type				
Rural	5490 (68.3)	18 927 (61.8)	2627 (68.4)	9847 (62.4)
Urban	2548 (31.7)	11 681 (38.2)	1214 (31.6)	5944 (37.6)
Geographical residence area in Tennessee				
East	2997 (37.3)	10 845 (35.4)	1410 (36.7)	5470 (34.6)
Middle	3420 (42.6)	12 425 (40.6)	1668 (43.4)	6463 (40.9)
West	1621 (20.2)	7338 (24.0)	763 (19.9)	3858 (24.4)

### Long-term Opioid Use

**Figure 2.**
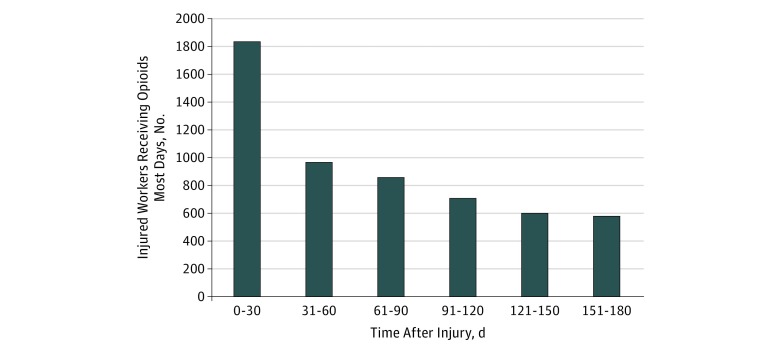
Number of Injured Workers Receiving Opioids Most Days for 180 Days After Injury

The number of injured workers who received an opioid on most days decreased sharply from 1834 to 966 between the first and second month after injury and decreased less steeply between 31 and 120 days after injury. The number plateaued after 120 days (Figure 2).

#### Derivation Model Analyses

In unadjusted analyses of the derivation group (Table 3), long-term opioid use was associated with receiving 20 or more days’ supply in the initial prescription compared with less than 5 days’ supply (OR, 14.00; 95% CI, 11.89-16.48), visiting 3 or more prescribers compared with visiting 1 (OR, 22.76; 95% CI, 19.55-26.51), and visiting 3 or more pharmacies compared with visiting 1 (OR, 18.82; 95% CI, 16.00-22.15). In the multivariable predictive model (see Table 3 for covariates), long-term opioid use continued to be associated with receiving 20 or more days’ supply in the initial prescription compared with less than 5 days’ supply (OR, 28.94; 95% CI, 23.44-35.72), and even 5 days’ to 9 days’ supply was associated with an increase in the odds of long-term use compared with less than 5 days’ supply (OR, 1.83; 95% CI, 1.56-2.14). Visiting 3 or more prescribers, compared with visiting 1, also continued to be associated with long-term opioid use (OR, 14.91; 95% CI, 12.15-18.29). Adding the number of prescribers visited to the model decreased the association of long-term opioid use with the number of pharmacies visited. Lower odds of long-term opioid use were found in finger injuries compared with other body parts (OR, 0.62; 95% CI, 0.46-0.84). Higher odds were found in rural residence compared with urban residence (OR, 1.51; 95% CI, 1.31-1.73), receiving a long-acting opioid within 30 days of injury compared with receiving only short-acting opioids (OR, 3.01; 95% CI, 2.16-4.20), receiving overlapping opioid and benzodiazepine prescriptions within 30 days of injury (OR, 1.38; 95% CI, 1.10-1.72), visiting 3 or more pharmacies for opioids compared with visiting 1 pharmacy (OR, 4.54; 95% CI, 3.70-5.56), and receiving 160 or more daily MME within 30 days of injury compared with less than 40 MME (OR, 3.24; 95% CI, 2.12-4.95) (Table 3). Receiving a long-acting opioid contributed to better model discrimination than comparing specific opioid formulations (C statistic, 0.92 vs 0.91).

**Table 3.** Associations of Demographic, Injury, and Opioid Use Variables With Long-term Opioid Use After Injury in 30 608 Injured Workers Who Reported 1 Injury to Tennessee Workers’ Compensation and Were Opioid Free at Time of Injury^a^^,^^b^

Characteristic	OR (95% CI)^c^	
	Unadjusted	Adjusted^d^
Part of body injured		
Other	1 [Reference]	1 [Reference]
Lower back	1.66 (1.43-1.92)	1.63 (1.38-1.93)
Finger(s)	0.43 (0.33-0.57)	0.62 (0.46-0.84)^e^
Residence type		
Urban	1 [Reference]	1 [Reference]
Rural	0.71 (0.63-0.80)	1.51 (1.31-1.73)
Initial days’ supply		
<5	1 [Reference]	1 [Reference]
5-9	1.75 (1.51-2.03)	1.83 (1.56-2.14)
10-19	3.80 (3.20-4.50)	4.73 (3.90-5.75)
≥20	14.00 (11.89-16.48)	28.94 (23.44-35.72)
Long-acting opioid within 30 d of injury	17.06 (13.16-22.11)	3.01 (2.16-4.20)
Overlapping opioid and benzodiazepine prescription days within 30 d of injury	3.80 (3.17-4.55)	1.38 (1.10-1.72)^e^
No. of prescribers visited for opioids within 90 d of injury		
1	1 [Reference]	1 [Reference]
2	5.25 (4.48-6.15)	4.28 (3.55-5.17)
≥3	22.76 (19.55-26.51)	14.91 (12.15-18.29)
No. of pharmacies visited for opioids within 90 d of injury		
1	1 [Reference]	1 [Reference]
2	5.62 (4.93-6.41)	1.89 (1.60-2.21)
≥3	18.82 (16.00-22.15)	4.54 (3.70-5.56)
Maximum MME received within 30 d of injury		
<40	1 [Reference]	1 [Reference]
40-159	2.69 (2.39-3.01)	1.67 (1.45-1.92)
≥160	8.18 (5.77-11.61)	3.24 (2.12-4.95)

Abbreviations: MME, morphine milligram equivalents; OR, odds ratio.

^a^ Long-term opioid use was defined as receiving an opioid on most days in the 90 days after injury.

^b^ Results from unconditional logistic regression analyses for derivation model (C statistic, 0.92).

^c^ *P* < .001 unless otherwise noted.

^d^ Adjusted for all variables in this Table.

^e^ *P* < .01.

#### Derivation and Validation Model Fit

The derivation model and validation model found similar associations for all variables, but the validation model did not have a significant association of overlapping opioid and benzodiazepine prescriptions with long-term opioid use (eTable 1 in the Supplement). Models had good discrimination, with C statistics from receiver operating characteristic curves of 0.92 in the derivation model and 0.91 in the validation model (eFigure 1 and eFigure 2 in the Supplement). Deviance statistics for the derivation (1194.77) and validation (652.47) models were significant at low *P* values, indicating a good fit (*P* = .001 and *P* = .009, respectively). Pearson statistics (1687.68 and 1067.39, respectively) were significant at *P* < .001, indicating good fit, and Hosmer-Lemeshow statistics (108.57 and 49.35, respectively) were significant, indicating good fit. When the number of prescribers and pharmacies visited were removed from the derivation model, discrimination decreased from a C statistic of 0.92 to 0.80. Associations were similar to the model with prescribers and pharmacies, except that the OR for an initial supply of 20 or fewer days decreased and the OR for amount of MME increased (eTable 4 in the Supplement).

Risk factors shown to be associated with opioid receipt in a previous study of this cohort^12^ were also evaluated for their association with long-term use. When the model was restricted to people with fractures, the model fit was lowered to a C statistic of 0.87, and the association of receiving 20 or fewer days’ initial supply decreased (adjusted OR, 6.30; 95% CI 3.84-10.18) but was still significant, while lower back injuries and overlapping opioid and benzodiazepine prescriptions were no longer significant (eTable 2 in the Supplement). Restricting the model to the most common injury (ie, strains, sprains, and tears) lowered the model fit to a C statistic of 0.91, while associations were unchanged except that finger injuries and MME of 160 or more were no longer significantly associated with long-term opioid use (eTable 3 in the Supplement). Hydrocodone and oxycodone were the most commonly prescribed opioids in both injury types and long-term use statuses (eTable 5 in the Supplement).

Dose escalation was observed in 431 patients (23.5%) who began long-term opioid use. The mean (SD; range) increase between the first and third months after injury was 34.04 (45.23; 0.22-1171.43) daily MME. Among injured workers who experienced dose escalation, 404 (69.4%) increased by more than 10 daily MME over 3 months, and 79 (13.6%) surpassed the Tennessee chronic pain guidelines threshold of 120 daily MMEs during this time. Among injured workers who qualified as having long-term use in the first 90 days after injury, 557 patients (31.5%) were still using opioids on most days 6 months after injury.

## Discussion

In this study, 6 opioid-use characteristics (days’ supply of first prescription, receipt of a long-acting opioid within 30 days of injury, overlapping opioid and benzodiazepine prescription, number of prescribers and pharmacies visited within 90 days of injury, and maximum MME received within 30 days of injury) were associated with long-term opioid use in previously opioid-free injured workers. The strongest association was receiving 20 or more days’ supply in the initial prescription, followed by visiting 3 or more prescribers for opioids, associations which have been found in other populations.^22^ Characteristics of opioid prescriptions, especially the initial prescription, had a greater association with long-term use than patient demographic or injury characteristics.

A previous study of this cohort^12^ estimated that the prevalence of receiving an opioid after injury was 22.8% within 1 week, 29.7% within 1 month, and 33.3% within 6 months. In this study, most workers (79.6%) who received opioids after injury were opioid free before injury, and 4.0% of these became long-term users. Notably, one-third of patients who qualified as long-term opioid users in the 90 days after injury continued to use opioids 180 days after injury. Although dose escalation was noted in only one-quarter of long-term users, it can contribute to opioid-related harms, and surpassing 50 MME per day can increase the risk of overdose by 30%.^10^ Since Tennessee law designates 30 days’ supply as the maximum that can be dispensed,^23^ opioid users had to have received at least 2 opioid prescriptions over at least 2 months to surpass 45 prescription days and meet the definition of a long-term user.

Although prescribing patterns were the strongest factors associated with long-term opioid use, this study also identified associations of part of body injured and rural residence. Whereas other studies have shown mixed associations of sex with long-term opioid use,^17,18^ no association of sex was found in this study. Like a 2018 study of WC claimants in Maryland,^5^ this study found an association of age with opioid use in bivariable analyses, but the inclusion of age decreased the performance of the predictive model. Other studies have shown mixed associations of age with long-term opioid use.^17,18,24^ Like the Maryland study,^5^ this study found a positive association of strains, sprains, and tears with long-term use. However, this study used a larger population (all injured workers vs only those who received WC medical care), focused on prescribing rather than patient characteristics, and measured more opioid prescriptions (all dispensed prescriptions vs only those paid for by employers’ insurance) in defining long-term use.

Addressing injury as a gateway to long-term opioid use is an important step toward curbing the opioid epidemic.^25^ Although previous studies have identified risk factors for the development of long-term opioid use, this appears to be the first to comprehensively measure all opioid prescriptions, regardless of insurance type, opioid dependence, or substance use disorder diagnosis, for long-term use in injured workers. In this predictive model, prescribing practices appear to be more associated with opioid use than demographic characteristics. Unlike patient demographic characteristics, prescribing may be modified to reduce patient risk.^26,27^

### Limitations

This study has limitations. Owing to unavailability of clinical data, this study was not able to consider several major factors associated with opioid use disorders, including genetic predisposition, mental illness, other drug use, race/ethnicity, and surgery status.^28^ However, our study provides an approach for using existing public health data sources to identify important factors associated with long-term opioid use in unique populations. Further, owing to a healthy worker effect where ill workers are excluded from employment, mental illness and other drug use may not cause much confounding in the model.^29^

Lack of data on comorbidities may also account for some of the differences seen in the model when the number of prescribers and dispensers visited were excluded. The number of contacts with medical care could indicate multiple health conditions, and medical comorbidities have been associated with long-term opioid use in other studies.^18^ Multiple contacts may also be an indication of drug-seeking behavior among people with an opioid use problem.^30^ Patient reports or flags in the CSMD about visiting multiple prescribers and pharmacies can still serve as a warning sign of potential long-term use.

The effect of race and ethnicity may be a larger concern; studies in other states show that white patients tend to be prescribed opioids more frequently^31^ and are more likely to overdose on opioids than black patients,^32^ but nonwhite workers are more likely to receive WC.^33^ Stratifying by race/ethnicity may provide more granular detail toward understanding the trends in the epidemic and directing resources toward groups that would benefit from increased attention. It may also decrease the association of residence type with opioid use, as white residents are more concentrated in rural areas of Tennessee.^25^ Although not all potential factors associated with long-term opioid use could be assessed, the variables included in this study resulted in a predictive model with extraordinarily good fit.

This study provides a good picture of the development of long-term opioid use in previously opioid-free injured workers in Tennessee but may not be generalizable to injured workers who do not report injuries to WC, report more than 1 injury, or live and work in other areas. Additionally, this study measures only prescriptions that were filled legally in Tennessee and may have missed prescriptions that were not consumed or were obtained from other sources. Misclassification of opioid-free patients owing to opioid supply from nonlegal sources or sources not measured by the CSMD is possible.

## Conclusions

To our knowledge, this was the first study to examine long-term opioid use in Tennessee and among the first nationally to use a prescription drug monitoring program to measure long-term opioid use in injured workers with prescription records instead of clinical or insurance records. Developing long-term use appears to be more associated with prescribing practices, especially days’ supply of the initial prescription, than patient or injury characteristics. These practices can be modified to reduce patient risk of overdose and associated morbidities.

## Notes

Supplement. **eTable 1.** Adjusted Odds of Long-term Opioid Use After Injury in Injured Workers Who Reported 1 Injury to Tennessee Workers’ Compensation and Were Opioid Free at the Time of Injury (n = 46 399) **eTable 2.** Adjusted Odds of Long-term Opioid Use After Injury in Injured Workers Who Reported 1 Injury to Tennessee Workers’ Compensation, Were Opioid Free at the Time of Injury, and Had a Fracture (n = 4351) **eTable 3.** Adjusted Odds of Long-term Opioid Use After Injury in Injured Workers Who Reported 1 Injury to Tennessee Workers’ Compensation, Were Opioid Free at the Time of Injury, and Had a Strain, Sprain, or Tear (n = 17 833) **eTable 4.** Adjusted Odds of Long-term Opioid Use After Injury in Injured Workers Who Reported 1 Injury to Tennessee Workers’ Compensation and Were Opioid Free at the Time of Injury (n = 30 608), With Number of Prescribers and Number of Pharmacies Removed From the Model **eTable 5.** Type of Drug Received in First Opioid Prescription After Injury, by Opioid-Free Status **eFigure 1.** Receiver Operating Characteristic (ROC) Curves of the Multivariable Unconditional Logistic Regression Derivation Model **eFigure 2.** Receiver Operating Characteristic (ROC) Curves of the Multivariable Unconditional Logistic Regression Validation Model
