# Supplementary material for: Prevalence and Risk Factors Associated With Long-term Opioid Use After Injury Among Previously Opioid-Free Workers
Source: JAMA Netw Open. 2019 Jul 17;2(7):e197222. doi: 10.1001/jamanetworkopen.2019.7222 (PMC6647548; doi:10.1001/jamanetworkopen.2019.7222)
Supplement: Supplement. — eTable 1. Adjusted Odds of Long-term Opioid Use After Injury in Injured Workers Who Reported 1 Injury to Tennessee Workers’ Compensation and Were Opioid Free at the Time of Injury (n = 46 399) eTable 2. Adjusted Odds of Long-term Opioid Use After Injury in Injured Workers Who Reported 1 Injury to Tennessee Workers’ Compensation, Were Opioid Free at the Time of Injury, and Had a Fracture (n = 4351) eTable 3. Adjusted Odds of Long-term Opioid Use After Injury in Injured Workers Who Reported 1 Injury to Tennessee Workers’ Compensation, Were Opioid Free at the Time of Injury, and Had a Strain, Sprain, or Tear (n = 17 833) eTable 4. Adjusted Odds of Long-term Opioid Use After Injury in Injured Workers Who Reported 1 Injury to Tennessee Workers’ Compensation and Were Opioid Free at the Time of Injury (n = 30 608), With Number of Prescribers and Number of Pharmacies Removed From the Model eTable 5. Type of Drug Received in First Opioid Prescription After Injury, by Opioid-Free Status eFigure 1. Receiver Operating Characteristic (ROC) Curves of the Multivariable Unconditional Logistic Regression Derivation Model eFigure 2. Receiver Operating Characteristic (ROC) Curves of the Multivariable Unconditional Logistic Regression Validation Model [file jamanetwopen-2-e197222-s001.pdf]

Supplementary Online Content

Durand Z, Nechuta S, Krishnaswami S, Hurwitz EL, McPheeters M. Prevalence and risk factors associated with long-term opioid use after injury among previously opioid-free workers. *JAMA Netw Open*. 2019;2(7):e197222. doi:10.1001/jamanetworkopen.2019.7222

eTable 1. Adjusted Odds of Long-term Opioid Use After Injury in Injured Workers Who Reported 1 Injury to Tennessee Workers' Compensation and Were Opioid Free at the Time of Injury (n = 46 399)

eTable 2. Adjusted Odds of Long-term Opioid Use After Injury in Injured Workers Who Reported 1 Injury to Tennessee Workers' Compensation, Were Opioid Free at the Time of Injury, and Had a Fracture (n = 4351)

eTable 3. Adjusted Odds of Long-term Opioid Use After Injury in Injured Workers Who Reported 1 Injury to Tennessee Workers' Compensation, Were Opioid Free at the Time of Injury, and Had a Strain, Sprain, or Tear (n = 17 833)

eTable 4. Adjusted Odds of Long-term Opioid Use After Injury in Injured Workers Who Reported 1 Injury to Tennessee Workers' Compensation and Were Opioid Free at the Time of Injury (n = 30 608), With Number of Prescribers and Number of Pharmacies Removed From the Model

eTable 5. Type of Drug Received in First Opioid Prescription After Injury, by Opioid-Free Status

eFigure 1. Receiver Operating Characteristic (ROC) Curves of the Multivariable Unconditional Logistic Regression Derivation Model

eFigure 2. Receiver Operating Characteristic (ROC) Curves of the Multivariable Unconditional Logistic Regression Validation Model

This supplementary material has been provided by the authors to give readers additional information about their work.

© 2019 Durand Z et al. *JAMA Network Open.*

eTable 1. Adjusted Odds of Long-term Opioid Use After Injury in Injured Workers Who Reported 1 Injury to Tennessee Workers' Compensation and Were Opioid Free at the Time of Injury (n = 46,399)

Characteristic	Validation model (n=15,791; c=0.91)		
	Adjusted OR	95% CI	
		lower	upper
Part of body injured			
Other	ref		
Lower back	1.5*	1.19	1.98
Finger(s)	0.5*	0.35	0.82
Residence type			
Rural	ref		
Urban	1.5**	1.22	1.82
Initial days' supply			
<5	ref		
5-9	1.7**	1.34	2.10
10-19	3.3**	2.45	4.47
≥20	24.8**	18.31	33.48
Long-acting opioid received within 30 days of injury	2.8**	1.68	4.62
Overlapping opioid and benzodiazepine prescription days within 30 days of injury	0.8	0.58	1.18
Number of prescribers visited for opioids within 90 days of injury			
1	ref		
2	4.8**	3.59	6.30
≥3	20.0**	14.91	26.94
Number of pharmacies visited for opioids within 90 days of injury			
1	ref		
2	1.9**	1.48	2.33
≥3	3.1**	2.27	4.21
Maximum MME[1] received within 30 days of injury			
<40	ref		
40-159	1.6**	1.28	1.92
≥160	3.4**	1.82	6.22

* p<0.01
** p<0.001
[1] MME = morphine milligram equivalents

© 2019 Durand Z et al. *JAMA Network Open.*

eTable 2. Adjusted Odds of Long-term Opioid Use After Injury in Injured Workers Who Reported 1 Injury to Tennessee Workers' Compensation, Were Opioid Free at the Time of Injury, and Had a Fracture (n = 4351)

Characteristic	Derivation model (n=2,755; c=0.87)			Validation model (n=1,596; c=0.85)		
	Adjusted OR	95% CI		Adjusted OR	95% CI	
		lower	upper		lower	upper
Part of body injured						
Other	ref			ref		
Lower back	2.0	0.63	6.23	4.4	0.95	19.91
Finger(s)	0.3*	0.10	0.70	0.6	0.24	1.61
Residence type						
Rural	ref			ref		
Urban	1.7**	1.17	2.46	1.9*	1.14	3.21
Initial days' supply						
<5	ref			ref		
5-9	1.7*	1.12	2.45	2.2*	1.29	3.72
10-19	6.3**	3.84	10.19	5.3**	2.70	10.47
≥20	11.5**	5.95	22.42	7.3*	2.62	20.54
Long-acting opioid within 30 days of injury	2.7**	1.38	5.29	2.5	0.98	6.14
Overlapping opioid and benzodiazepine prescription days within 30 days of injury	1.2	0.61	2.17	0.8	0.30	2.05
Number of prescribers visited for opioids within 90 days of injury						
1	ref			ref		
2	4.2**	2.46	7.12	2.9*	1.45	5.90
≥3	12.0**	6.76	21.22	9.3**	4.46	19.20
Number of pharmacies visited for opioids within 90 days of injury						
1	ref			ref		
2	1.2	0.81	1.83	1.7*	1.03	2.91
≥3	3.5**	2.20	5.69	3.4*	1.58	7.29
Maximum MME[2] received within 30 days of injury						
<40	ref			ref		
40-159	1.5*	1.003	2.33	1.6	0.93	2.84
≥160	4.5**	2.22	8.95	2.9	0.94	8.68

* p<0.05
** p<0.001
[1] Long-term opioid use was defined as receiving an opioid on most days in the 90 days after injury
[2] MME = morphine milligram equivalents

© 2019 Durand Z et al. *JAMA Network Open.*

eTable 3. Adjusted Odds of Long-term Opioid Use After Injury in Injured Workers Who Reported 1 Injury to Tennessee Workers' Compensation, Were Opioid Free at the Time of Injury, and Had a Strain, Sprain, or Tear (n = 17 833)

Characteristic	Derivation model (n=11,994; c=0.91)			Validation model (n=5,839; c=0.91)		
	Adjusted OR	95% CI		Adjusted OR	95% CI	
		lower	upper		lower	upper
Part of body injured						
Other	ref			ref		
Lower back	1.4*	1.14	1.71	1.4	1.00	1.84
Finger(s)	0.5	0.12	2.37	1.4	0.31	6.02
Residence type						
Rural	ref			ref		
Urban	1.5**	1.22	1.83	1.2	0.90	1.63
Initial days' supply						
<5	ref			ref		
5-9	1.4*	1.14	1.84	1.8*	1.26	2.53
10-19	3.8**	2.89	5.09	3.6**	2.29	5.70
≥20	24.8**	18.20	33.69	25.0**	15.72	39.77
Long-acting opioid within 30 days of injury	4.2**	2.41	7.27	4.9*	1.91	12.35
Overlapping opioid and benzodiazepine prescription days within 30 days of injury	1.5 (p=0.018)	1.07	2.01	1.1	0.64	1.77
Number of prescribers visited for opioids within 90 days of injury						
1	ref			ref		
2	4.4**	3.34	5.83	5.7**	3.71	8.62
≥3	14.9**	11.00	20.09	23.5**	14.97	36.86
Number of pharmacies visited for opioids within 90 days of injury						
1	ref			ref		
2	2.0**	1.61	2.57	1.9*	1.37	2.75
≥3	5.5**	4.10	7.47	2.4*	1.44	3.86
Maximum MME[2] received within 30 days of injury						
<40	ref			ref		
40-159	1.7**	1.36	2.08	1.3	0.97	1.83
≥160	1.8	0.61	5.08	6.4*	1.61	25.05

* p<0.01
** p<0.001
[1] Long-term opioid use was defined as receiving an opioid on most days in the 90 days after injury
[2] MME = morphine milligram equivalents

© 2019 Durand Z et al. *JAMA Network Open.*

eTable 4. Adjusted Odds of Long-term[1] Opioid Use After Injury in Injured Workers Who Reported 1 Injury to Tennessee Workers' Compensation and Were Opioid-Free at the Time of Injury (n = 30 608), With Number of Prescribers and Number of Pharmacies Removed From the Model

Characteristic	Derivation model (n=30,608; c=0.80) Adjusted OR	Derivation 95% CI lower	Derivation 95% CI upper	Validation model (n=15,791; c=0.78) Adjusted OR	Validation 95% CI lower	Validation 95% CI upper
Part of body injured						
Other	ref			ref		
Lower back	1.6**	1.40	1.92	1.7**	1.32	2.10
Finger(s)	0.5**	0.36	0.64	0.5*	0.36	0.81
Residence type						
Rural	ref			ref		
Urban	1.4**	1.20	1.55	1.4*	1.13	1.65
Initial days' supply						
<5	ref			ref		
5-9	1.8**	1.57	2.11	1.7**	1.35	2.07
10-19	3.9**	3.25	4.66	2.8**	2.15	3.76
≥20	15.6**	12.97	18.65	12.8**	9.85	16.63
Long-acting opioid within 30 days of injury	5.2**	3.87	7.10	4.5**	2.82	7.22
Overlapping opioid and benzodiazepine prescription days within 30 days of injury	1.9**	1.55	2.33	1.3	0.95	1.82
Maximum MME[3] received within 30 days of injury						
<40	ref			ref		
40-159	3.5**	3.09	3.99	3.4**	2.79	4.04
≥160	8.8**	5.92	13.21	10.2**	5.80	18.08

* p<0.01
** p<0.001
[1] Long-term opioid use was defined as receiving an opioid on most days in the 90 days after injury
[2] Results from unconditional logistic regression analyses for derivation model (c=0.92)
[3]MME = morphine milligram equivalents

eTable 5. Type of Drug Received in First Opioid Prescription After Injury, by Opioid-Free Status

	**Fracture (N=4,351)**				**Strain, sprain, or tear (N=17,833)**			
	**Long-term opioid use**				**Long-term opioid use**			
	**No (n=4,057)**		**Yes (n=294)**		**No (n=16,985)**		**Yes (n=848)**	
Drug	n	%	n	%	n	%	n	%
Hydrocodone short-acting	2487	61.30	127	43.20	10284	60.55	512	60.38
Oxycodone short-acting	1210	29.82	143	48.64	2021	11.90	117	13.80
Tramadol short-acting	314	7.74	17	5.78	4123	24.27	212	25.00
Codeine	54	1.33	1	0.34	544	3.20	11	1.30
Morphine long-acting	13	0.32	8	2.72	7	0.04	2	0.24

Note: Drug categories are nonexclusive

© 2019 Durand Z et al. *JAMA Network Open.*

eFigure 1. Receiver Operating Characteristic (ROC) Curves of the Multivariable Unconditional Logistic Regression Derivation Model

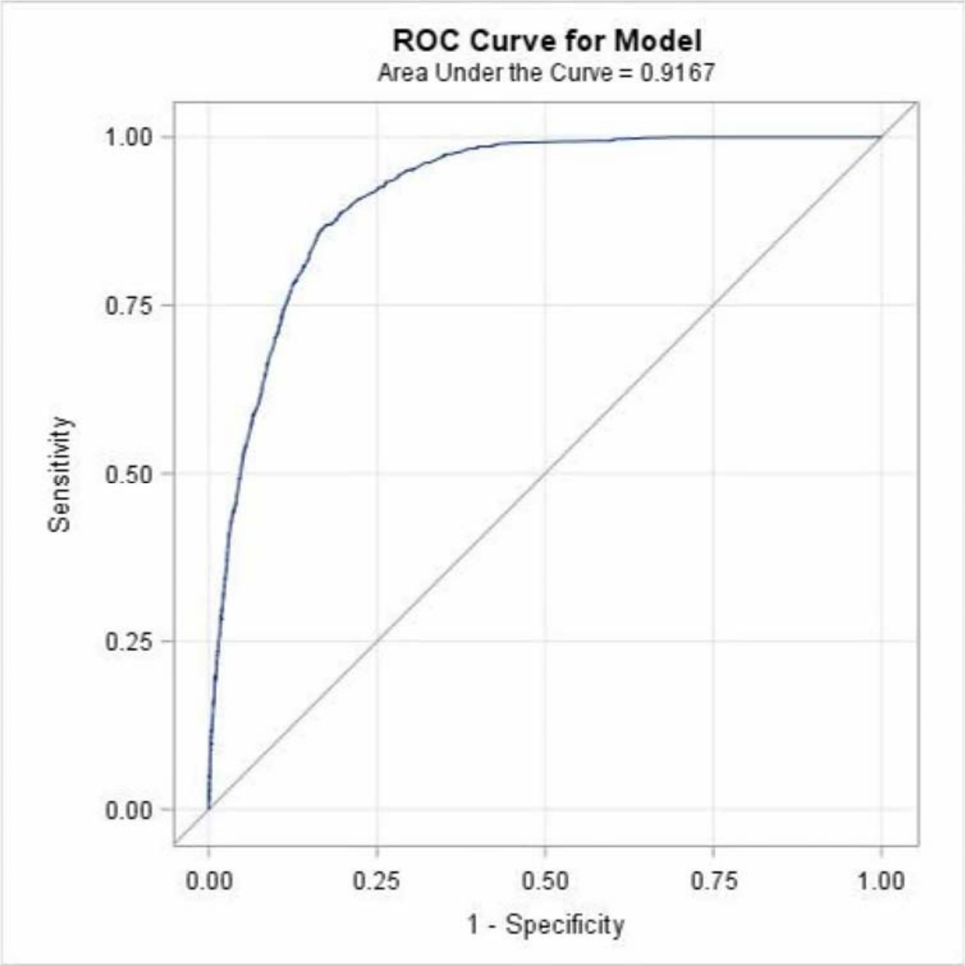

© 2019 Durand Z et al. *JAMA Network Open.*

eFigure 2. Receiver Operating Characteristic (ROC) Curves of the Multivariable Unconditional Logistic Regression Validation Model

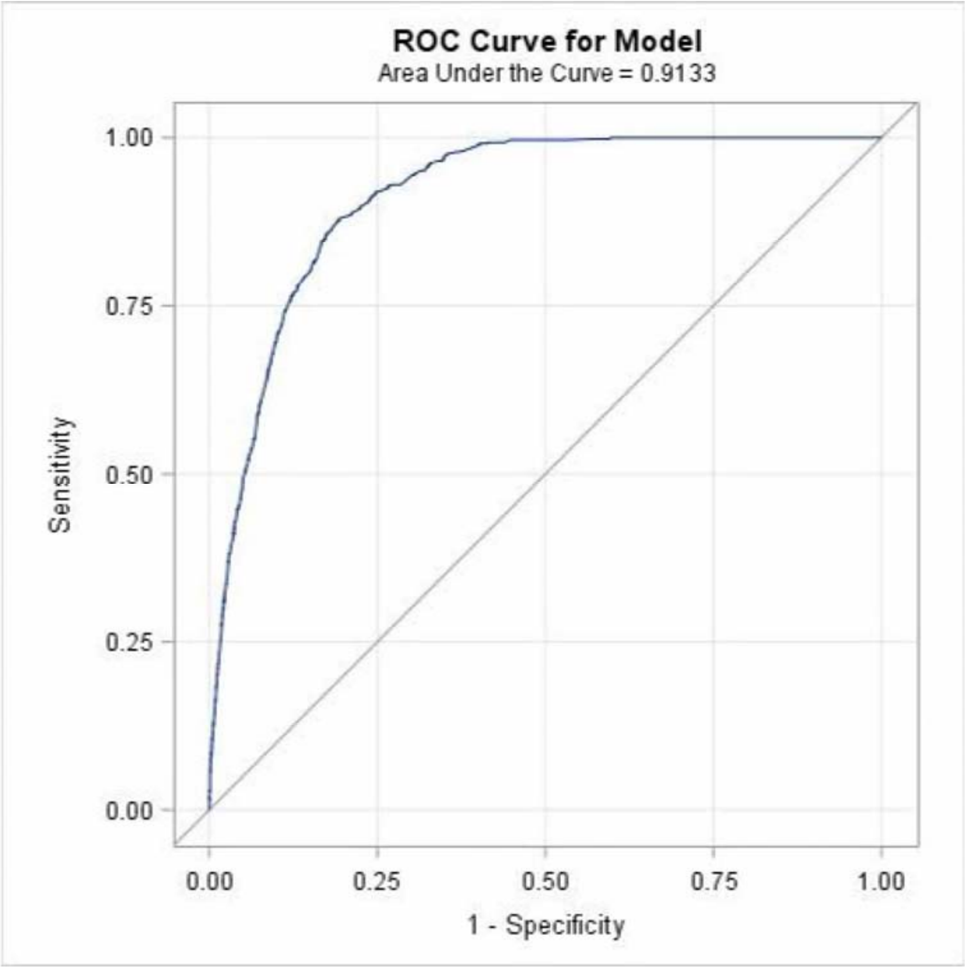

© 2019 Durand Z et al. *JAMA Network Open.*
